# Tigecycline in the treatment of severe pneumonia caused by *Chlamydia psittaci*: A case report and literature review

**DOI:** 10.3389/fmed.2022.1040441

**Published:** 2022-11-24

**Authors:** Jiaming Liu, Yuan Gao

**Affiliations:** Department of Pulmonary and Critical Care Medicine, Shengjing Hospital of China Medical University, Shenyang, China

**Keywords:** *psittaci*, *Chlamydia psittaci* pneumonia, severe pneumonia, tigecycline, metagenomic nextgeneration sequencing (mNGS)

## Abstract

Psittacosis is a zoonotic disease caused by *Chlamydia psittaci*. Systemic infections are mainly transmitted through the respiratory tract. The most common related disease is human atypical pneumonia, which is a rare pathogen of community-acquired pneumonia. Due to the difficulty of diagnosis, there have been few reports of *C. psittaci* pneumonia in the past. In recent years, with the widespread application of metagenomic next-generation sequencing (mNGS), the number of reported cases of *C. psittaci* has increased year by year. However, at present, most hospitals have little understanding of *C. psittaci*, especially for severe patients, and lack experience in diagnosis and treatment. Herein, we report the case of a 71-year-old woman with severe pneumonia that caused by *C. psittaci*. This patient was diagnosed through mNGS and was treated with tigecycline successfully. The level of IL-6 in the BALF was significantly increased. We discontinued tigecycline after mNGS of the blood was negative. In this review, we analyzed 53 cases to summarize the etiology, clinical manifestations, diagnosis and treatment strategies of severe *C. psittaci* pneumonia and hope to raise clinicians’ awareness of this disease.

## Introduction

Psittacosis is a zoonotic disease caused by *Chlamydia psittaci* (1). Humans are infected through the respiratory tract, and the most common related disease is human atypical pneumonia (2). It is a rare cause of community-acquired pneumonia, accounting for approximately 1% of community-acquired pneumonia. Some patients with psittacosis progress rapidly and can die if not treated in time. Due to the difficulty of diagnosis, there have been few reports of related cases in the past. In recent years, due to the widespread use of metagenomic next-generation sequencing (mNGS), reports of *C. psittaci* have been increasing year by year. This article reports a case of severe *C. psittaci* pneumonia who was diagnosed by mNGS and improved after treatment with tigecycline. There have been few cases of using tigecycline to treat *C. psittaci* pneumonia in the past, so it is necessary to summarize this case and review the literature.

## Case presentation

**FIGURE 1:**
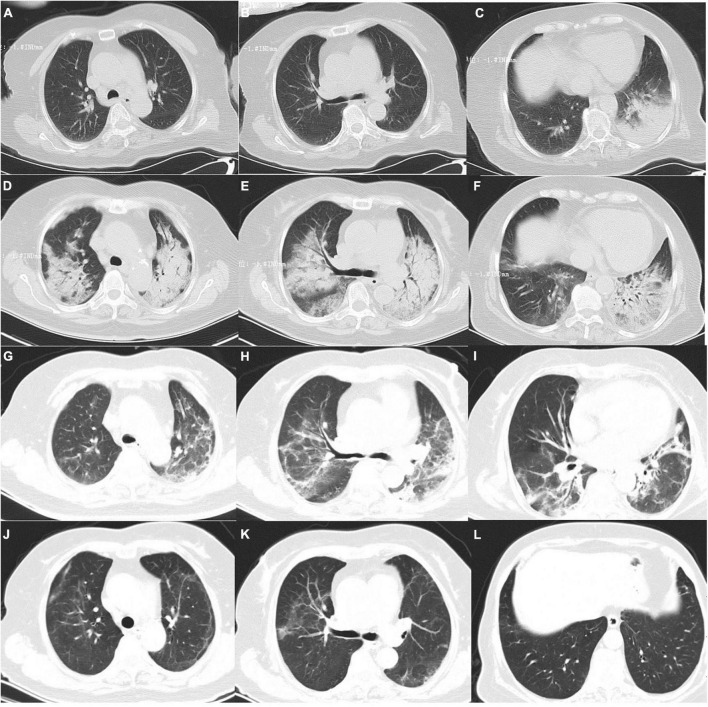
Patient’s chest CT scans: panels **(A–C)** show CT scans at the time of the patient’s first day of illness (November 7) in the local hospital. Panel **(D–F)** show CT scans on the 10th day of illness (November 17) in the local hospital. Panel **(G–I)** show CT scans on the 10th day after admission (November 27). Panel **(J–L)** show CT scans on the 26th day after admission (December 12).

The patient of a 71-year-old woman was admitted to our hospital because of fever for 10 days and dyspnea for 5 days on November 17, 2020. She presented with fever and chills 10 days before admission, and the maximum body temperature was 40°C, accompanied by fatigue and dry cough without sputum. Chest CT at the local hospital showed pneumonia in the inferior lobe of the left lung and mediastinal lymph node enlargement (Figure 1). Routine blood examination showed that the white blood cell count was 10.2 × 10^9^/L and the CRP level was 139 mg/L. She went to a local clinic and applied cephalosporin antibiotics, but the above symptoms did not improve. Nausea and vomiting occurred 7 days prior, accompanied by diarrhea and watery stools, without abdominal pain. Then, she was hospitalized in the local hospital. Using ceftriaxone and moxifloxacin intravenously for 7 days showed no improvement. Dyspnea occurred 5 days prior and was aggravated after the event. One day before admission, the patient’s dyspnea was significantly aggravated, and re-examination of chest CT was significantly worse than before (Figure 1). The patient was treated with tracheal intubation and ventilator-assisted ventilation. She was sent immediately to Shengjing Hospital of China Midical University and was hospitalized in the Department of Pulmonary and Critiacl Care Medicine.

The patient suffered from coronary atherosclerotic cardiopathy for 3 years, and she took aspirin intermittently. She had no hypertension and diabetes. She had no hepatitis and tuberculosis. She resides in Liaoning Province, northern China. She is a retired employee, has no smoking and drinking habits, and has no drug or food allergy.

When the patient was admitted to our hospital (day 1), her vital signs were as follows: body temperature 36.5°C, pulse rate 80 beats/min, respiratory rate 27 beats/min, blood pressure 117/69 mmHg, and pulse oxygen saturation 96% with a fraction of inspired oxygen (FiO_2_) of 0.80. Conscious, no yellowing of skin and sclera, no paleness of eyelid conjunctiva. Breath sounds in both lungs were rough, and wet rales were heard on auscultation. There was no audible murmur on cardiac auscultation. Tenderness in the abdomen and hepatosplenomegaly were not detected. No edema of both lower limbs.

The laboratory dates after admission to our hospital were as follows: arterial blood gas analysis showed a pH of 7.513, PaO_2_ of 98.6 mmHg, PaCO_2_ of 28.2 mmHg, HCO_3_- of 22.5 mmol/L and oxygenation index of 123.2. The white blood cell count was 18.5 × 10^9^/L, with an elevated neutrophil ratio of 90.3%. The concentration of C-reactive protein (CRP) was 253.7 mg/L. The concentrations of procalcitonin (PCT, normal < 0.05 ng/ml) and interleukin-6 (IL-6, normal ≤5.4 pg/ml) were 2.91 ng/ml and 12.64 pg/ml, respectively. The D-dimer (normal 0–252 μg/L) was 2161 μg/L. Albumin level (normal 35–53 g/L) was 23.0 g/L. Alanine aminotransferase (ALT, normal 0–40 U/L) was 68 U/L, and aspartate aminotransferase (AST, normal 5–34 U/L) was 69 U/L. Bilirubin, creatinine, and electrolytes were within normal limits. The creatine kinase (CK, normal 29–200 U/L) was 75 U/L, and the creatine kinase isoenzyme (CK-MB, normal 0–24 U/L) was 17.4 U/L. The concentrations of B-type natriuretic peptide (BNP, normal 0–154.7 pg/mL) and cardiac troponin I (cTnI, normal 0–0.0116 U/L) were 300.8 pg/ml and 0.7743 μg/L, respectively. Mycoplasma pneumoniae IgM was positive, and IgG was negative. Chlamydia pneumoniae IgM was negative, and IgG was positive. The Epstein Barr virus DNA quantification was 8.15E + 03 copies/ml (normal < 1.0E + 03 copies/ml). Tests for cytomegalovirus, influenza A virus, influenza B virus, aenovirus and legionella bacteria were all negative. The β-D-glucan test and galacto Mannan test were both negative. T-SPOT-T and Tuberculin test were negative. The indicators for autoimmune diseases (anti-extractable nuclear antigen antibody, anti-nuclear antibody and anti-neutrophilic cytoplasmic antibody) were negative. The numbers of CD3 (normal 690–2,540/μl), CD8 (normal 190–1,140/μl) and CD4 (normal 410–1,590/μl) T lymphocytes in blood were 254, 41, and 165/μl, respectively. The concentrations of carcinoembryonic antigen (CEA, normal 0–5 ng/ml), cytokeratin 19 fragment (CYFRA21-1, normal 0.1–3.3 ng/ml) and neuron-specific enolase (NSE, normal 0–16.3 ng/ml) were 2.37, 6.30, and 17.32 ng/ml, respectively.

**FIGURE 2:**
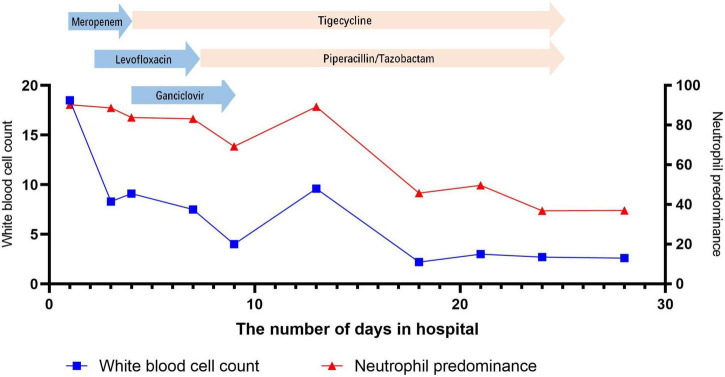
Change in white blood cell count and neutrophil predominance during hospitalization at the Shengjing Hospital of China Medical University.

**FIGURE 3:**
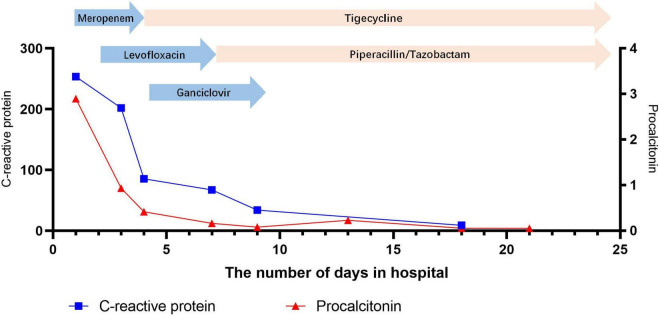
Change in C-reactive protein and procalcitonin during hospitalization at the Shengjing Hospital of China Medical University.

**FIGURE 4:**
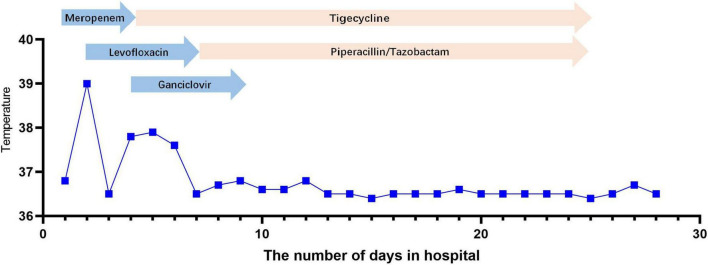
Body temperature and antimicrobial treatment during hospitalization at the Shengjing Hospital of China Medical University.

After admission, the patient continued to be treated with ventilator-assisted ventilation. Empirical anti-infective treatments, including meropenem, levofloxacin, ganciclovir, and arbidol, were used initially. After the initial treatment, the patient’s peak temperature decreased, but she still had fever. On the second day of admission (November 18), the patient underwent bronchoscopic alveolar lavage examination, and bronchoalveolar lavage fluid (BALF) was sent to Nanjing Difei Medical Laboratory Co., Ltd. for mNGS examination. The concentration of interleukin-6 (IL-6) in the BALF was 4,519.04 pg/ml (normal range 0–5.4 pg/ml). On the 4th day of admission (November 20), the result of mNGS of the BALF was reported, and 70 sequence reads corresponding to *C. psittaci* were identified and there was no sequence read corresponding to other pathogens. The patient had no history of direct contact with birds and poultry before the illness, but there were neighbors raising pigeons in the residential area, not except for indirect contact. According to the results of mNGS of BALF suggesting *C. psittaci* infection (Table 1), antibacterial agents were switched to tigecycline (100 mg intervenous drop infusion on day 4, then 50 mg q12 h intervenous drop infusion on days 4–25) combined with levofloxacin (0.5 g qd intervenous drop infusion, days 2–7), and meropenem was discontinued. The patient’s blood sample was sent to Guangzhou Weiyuan Gene Technology Co., Ltd. for mNGS testing again, and the results of the blood samples still identified 38 sequence reads corresponding to *C. psittaci* (Table 1). She was diagnosed with *C. psittaci* pneumonia. Her body temperature returned to normal on the 6th day of admission (Figures 1–4), and her dyspnea gradually eased. On the 7th day of admission (November 23), the patient’s sputum culture revealed Pseudomonas aeruginosa and Candida albicans. Considering the patient had a secondary infection. According to the results of drug sensitivity, the patient was administered piperacillin tazobactam (4.5 g q8 h intervenous drop infusion, days 8–25) while strengthening oral care. The patient had sinus bradycardia, and the electrocardiogram showed a prolonged QT interval, so levofloxacin was ceased. On the 10th day of admission (November 26), the patient’s tracheal intubation was removed. Re-examination of chest CT showed that lung inflammation was significantly absorbed (Figure 1). On the 24th day of admission (December 10), blood mNGS was performed again and was negative for *C. psittaci* (Table 1). Tigecycline and piperacillin tazobactam were discontinued. Re-examination of the chest CT (December 12) showed that the inflammation was well absorbed, and only a few fiber cord shadows were left. On the 29th day of admission (December 15), the patient was discharged from the hospital, and her status was close to premorbid condition.

**TABLE 1:** The patient’results of the mNGS of BALF and blood.

	The mNGS of BALF (November 19)	The NSG of blood (November 20)	The NSG of blood (December 10)
*Chlamydia psittaci* (reads)	70	38	–
*Propionibacterium acnes* (reads)	–	32	478
*Moraxella osloensis* (reads)	–	60	–
*Staphylococcus hominis*(reads)	–	6	105
*Staphylococcus warneri* (reads)	–	–	592
*Malassezia globosa* (reads)	–	–	4

Other bacteria, including *Propionibacterium acnes*, *Moraxella osloensis*, *Staphylococcus hominis*, *Staphylococcus warneri*, and *Malassezia globose*, were also detected by mNGS in the patient’s blood samples (Table 1). These bacteria are all skin microecological flora and not considered as pathogenic bacteria.

## Literature review

We searched the PubMed database for articles on *C. psittaci* published before September 31, 2021. The search strategy was “*Chlamydia psittaci*” or “*Chlamydia psittaci* pneumonia”, and a total of 870 articles were found. Among them, there were 23 articles about severe *C. psittaci* infection, and 54 severe *C. psittaci* pneumonia patients were reported; 27 cases were treated with ventilators, and 7 cases died. A summary of the detailed information is shown in Table 2. Most cases had a history of direct or indirect exposure. Contact history included raising parrots, birds and poultry, etc., or going to a bird shop, passing through a poultry market. There were also a few patients who had been infected due to contact with pets or aborted sheep. There were also reports of human-to-human transmission. Most patients had underlying diseases. There were 2 pregnant women, and 1 of them died. Chest radiography or CT showed consolidation or ground glass-like changes, and some patients had pleural effusion.

**TABLE 2:** Summary of case series and case report of severe *C. psittaci* pneumonia.

Author, reported time	Reported area	Number of cases	Methods	Anti-infective drugs	History of exposure to avian or poultry	Clinical outcome
1. Chen et al. (31)	China	9	mNGS	The initial treatment of β-lactam and quinolones was ineffective. Changed to minocycline after diagnosis.	7 patients were in contact with ducks, pigeons, or poultry.	8 patients improved, 1 patient died.
2. Wang et al. (32)	China	3	mNGS	The initial treatment of carbapenems and quinolones was not effective. Changed to moxifloxacin/ceftriaxone combined with levofloxacin/moxifloxacin combined with doxycycline after diagnosis.	Bird or poultry contact	Improved
3. Zhang et al. (21)	China	1	mNSG	Doxycycline	May be related to the work environment of the wholesale market	Improved
4. Katsura et al. (33)	Japan	1	PCR	Meropenem	parrot	Died
5. Kong et al. (30)	China	2	mNGS	The initial treatment of moxifloxacin was not effective, changed to tigecycline was effective.	1 patient’s workplace was located upstairs of a farmers’ market (including poultry market areas). 1 patient had an exposure history of pigeon feces.	Improved
6. Shi et al. (12)	China	1	mNGS	The initial treatment of moxifloxacin was ineffective, changed to doxycycline + moxifloxacin + azithromycin after diagnosis.	No	Improved
7. Zhang et al. (34)	China	1	mNGS	The initial treatment of β-lactam and moxifloxacin was ineffective. Changed to moxifloxacin and doxycycline after diagnosis.	No	Improved
8. Yuan et al. (35)	China	1	mNGS	Meropenem + Doxycycline	Poultry	Improved, but died after discharge.
9. Meijer et al. (13)	Netherlands	1	PCR	Doxycycline, Ceftriaxone, Ciprofloxacin	Pigeons	Improved
10. Wu et al. (29)	China	13	mNGS	The initial treatment of β-lactams and quinolones was ineffective, changed to tetracyclines after the diagnosis.	Yes	11 patients improved, 2 patients died.
11. Teng et al. (36)	China	5	mNGS	1 patient received moxifloxacin; 2 patients received doxycycline; 2 patients received doxycycline combined with moxifloxacin.	3 patients had an exposure history of poultry.	4 patients improved, a pregnant woman died.
12. Wen et al. (28)	China	2	mNGS	1 patient was used ceftazidime, ofloxacin, ertapenem, micafungin, imipenem, tigecycline, moxifloxacin, and sulfamethoxazole, and changed to doxycycline combined with moxifloxacin after diagnosis. 1 patient used third-generation cephalosporin, moxifloxacin and peramivir, and changed to moxifloxacin after diagnosis.	1 patients had an exposure history of pigeons	Improved
13. Qi et al. (37)	China	1	mNGS	Piperacillin, imipenem, and moxifloxacin were ineffective. Changed to moxifloxacin and imipenem after diagnosis.	Duck	Improved
14. Arenas-Valls et al. (38)	Spain	2	PCR and MIF	Ceftriaxone, Levofloxacin, Doxycycline	Birds	Improved
15. Spoorenberg et al. (4)	Netherlands	1	PCR and MIF	Cephalosporins, Quinolones, Erythromycin	Pigeons	Improved
16. Fraeyman et al. (39)	Belgium	2	PCR	Moxifloxacin	Pigeons	Improved
17. Bourne et al. (40)	England	1	MIF	The initial treatment of ciprofloxacin was ineffective, and changed to erythromycin combined with rifampicin after diagnosis.	Budgerigar	Improved
18. Soni et al. (41)	Australian	2	CF and MIF	The initial treatment of ceftriaxone + erythromycin was ineffective, and changed to rifampicin + ciprofloxacin/ doxycycline after diagnosis.	Parrot	Improved
19. Verweij et al. (42)	Netherlands	1	PCR	The initial treatment of amoxicillin + erythromycin was ineffective, and changed to doxycycline + gentamicin after diagnosis.	Parrot	Improved
20. Lamáury et al. (10)	The United States	1	MIF	Used erythromycin, and changed to doxycycline after diagnosis.	Pigeons	Died
21. Shapiro et al. (43)	The United States	1	ulture + MIF	Used cefaclor, erythromycin, vancomycin, gentamicin, and ceftazidime. and changed to doxycycline after diagnosis.	Parrot	Improved
22. Villemonteix et al. (44)	France	1	CF and MIF	Amoxicillin + Clavulanic acid + Gentamicin + Spiramycin	Aborted sheep	Improved
23. Wang et al. (45)	China	1	mNGS	Meropenem and Doxycycline	Yes	Improved

Forty-one patients (54 cases in total) were treated with quinolones, including moxifloxacin, levofloxacin, and ciprofloxacin. Among them, 6 patients were improved by quinolones alone after diagnosis, and 29 patients were initially ineffective with quinolones. A total of 9 patients had been treated with macrolides, including azithromycin, erythromycin, and spiramycin. Five patients had no effect on initial treatment with macrolides. There was no successful case of using macrolides alone in severe patients. A total of 27 patients were treated with tetracycline (alone or in combination with quinolones and macrolides) after being diagnosed with psittacosis, including doxycycline and minocycline. Among them, 5 patients died of ineffective treatment, 1 patient improved but died after being discharged from the hospital, and the rest were cured. One pregnant patient had only used meropenem during the treatment and died. In 2 patients, the initial application of quinolones was not effective but improved after changing to tigecycline.

## Discussion

*Chlamydia psittaci* is an intracellular parasite and a gram-negative spherical pathogen that is the pathogen of epidemic avian chlamydia (1). The most common related disease is human atypical pneumonia, which is a rare cause of community-acquired pneumonia, accounting for approximately 1% of community-acquired pneumonia (2). A study on the detection rate of IgM antibodies to Mycoplasma and Chlamydia in infants and young children in Japan found that the positive rate of *C. psittaci* was approximately 2.2% (3). Spoorenberg et al. conducted PCR to detect *C. psittaci* in patients with community-acquired pneumonia in two hospitals in the Netherlands and found that the incidence of *C. psittaci* was much higher than that previously reported, approximately 4.8% (4). Among patients with severe pneumonia in the ICU, the incidence of *C. psittaci* could be as high as 8% (5). These findings suggest that the estimated incidence of *C. psittaci* in current epidemiological data may be underestimated, especially in critical patients.

The typical epidemiological data of *C. psittaci* are that humans are infected through contact with the eyes, beaks or intestinal feces of birds (1). Poultry (such as chickens, ducks, etc.) can also be infected with *C. psittaci* and then infect humans. The transmission routes include raising poultry or frequently visiting poultry markets (6). There were also reports in the that humans were infected through mammals, such as sheep and horses, but were rare (7, 8). There have also been reports of human-to-human transmission (9). Our patient had no history of direct contact with birds and poultry before the illness, but there were neighbors who raised birds in the patient’s residential area, not except for indirect contact.

The manifestations of *C. psittaci* include fever, headache, muscle aches, dry cough, dyspnea, etc., and some patients may have relative bradycardia. Severe cases may also involve other organs, including the heart, liver, skin, and central nervous system, and cause symptoms (10–14). Our patient had bradycardia, a prolonged Q-T interval, and elevated myocardial enzymological markers, which may be associated with *C. psittaci* involving the heart.

In most patients, the white blood cell counts were normal, the proportion of neutrophils was increased, the lymphocytes were decreased, C-reactive proteins (CRP) and erythrocyte sedimentation rates (ESR) were significantly increased, and the increase in procalcitonins was not obvious. In our patient, the initial white blood cell count did not increase significantly, but the CRP level increased significantly. After admission, the patient’s white blood cell count, CRP and procalcitonin were all increased significantly, which might be related to secondary infection (sputum culture suggests Pseudomonas aeruginosa).

Interleukin 6 is the main mediator of physiological, hematological, and immunological reactions during the acute phase of inflammation, especially regulating the synthesis of liver proteins in the acute phase. In rodent models of experimentally induced fever, the important role of IL-6 as a circulating endogenous pyrogen has been well established (15). Prohl et al. confirmed that after inoculating *C. psittaci* into the lungs of calves, the activity of IL-6 in BALF was significantly higher than that in blood serum and revealed no apparent relation between IL-6 in blood and body temperature, but they did reveal a relation between IL-6 and other markers of inflammation in BALF and concluded that a local inflammatory response in the lungs of infected calves caused fever (16). In our patient, IL-6 was also significantly increased in the BALF and was not significantly increased in the blood. This indicates that human infection with *C. psittaci* can also cause a strong local inflammatory response in the lungs and cause fever. The inflammatory response in the blood is mild and may have no relation with fever.

Chest CT of *C. psittaci* pneumonia showed ground glass-like changes or consolidation, and the lower lobes of the lung were often involved. This was occasionally accompanied by pleural effusion and mediastinal lymph node enlargement. Large shadows on the lung lobes and extensive bilateral pneumonia may also appear in severe cases (17). Chest CT of our patient showed consolidation of the lower lobe of the left lung initially and quickly progressed to extensive bilateral pneumonia, with hilar node enlargement, without pleural effusion. After the treatment was effective, the lung inflammation was significantly absorbed, only a few fiber cords remained, and the mediastinal lymph nodes were smaller than before.

There have been few reports of *C. psittaci* pneumonia in the past, on the one hand due to its low incidence and on the other hand due to the difficulty of diagnosis. Traditional pathogen culture is time-consuming, and *C. psittaci* needs to be cultured in cells, which requires very high laboratory conditions, so clinical application is rare. Serological testing is mainly used for retrospective studies, which has little value for the early diagnosis of severe patients, and most hospitals in China do not carry out such inspection items at present. PCR tests can quickly identify *C. psittaci* (18, 19), but this test has also not been carried out in Chinese hospitals. mNGS can quickly and accurately identify pathogens and has been widely used in the diagnosis of infectious diseases, especially to detect pathogens that cannot be detected by traditional methods (20). In recent years, with the widespread use of mNGS, an increasing number of cases of psittacosis have been diagnosed. We reviewed 23 articles, including 54 cases of severe Chlamydia psitsiti pneumonia. There were 12 articles using mNGS to confirm the diagnosis, all of which were reported in China.

In our patient, only *C. psittaci* was found in mNGS of BALF, with a sequence of 70 reads and without other background pathogens. We sent blood samples to other testing institutions for mNGS, and *C. psittaci* was also detected, with a sequence of 38 reads. These results indicate that the invasion of *C. psittaci* into the human body can not only cause lung infection but can also spread in the patient’s body and lead to explosive systemic disease, which may be related to the special infection mechanism of the intracellular bacteria. It has also been reported in the literature that *C. psittaci* first enters the reticuloendothelial cells of the liver and spleen to proliferate and then enters the lungs and other organs through the bloodstream (21, 22). Therefore, human psittacosis is a systemic infection, mainly respiratory infections (13, 23). Our patient was diagnosed with *C. psittaci* pneumonia. After symptomatic anti-infection treatment, the patient’s fever and dyspnea improved significantly, and the inflammatory indicators returned to normal. Re-examination of lung CT indicated that the lung inflammation was significantly absorbed. On the 24th day of hospitalization, we reexamined the patient’s blood mNGS, indicating that *C. psittaci*s was not detected. Then, tigecycline was discontinued, and the total course of treatment was 21 days. During the follow-up, the patient was in good condition and returned closely to her premorbid condition. Currently, the specific course of treatment for *C. psittaci* is uncertain and is generally believed to be 10–21 days. Whether a prolonged course of treatment can prevent recurrence is still controversial. Whether the negative results of mNGS in BALF and blood can be used as evidence of drug withdrawal remains to be further clinically observed.

Tetracyclines are the first choice for the treatment of *C. psittaci* pneumonia, including doxycycline, tetracycline and minocycline. In the previous articles about severe *C. psittaci* infection, 22 patients (54 cases in total) responded to tetracycline therapy in combination with or alone after diagnosis, and 5 patients did not respond to the treatment and died. The deaths were thought to be related to the subsequent infections of other resistant bacteria, but the possibility of tetracycline resistance has not been determined.

When the use of tetracycline is contraindicated, macrolides (erythromycin, azithromycin, etc.) can be used instead, but it may not be effective in patients with severe disease or pregnancy (24). In previous studies, there were successful cases of using macrolides alone, but they were all mild patients. There was no successful case of severe *C. psittaci* pneumonia treated with macrolides alone. These results indicate that macrolides have a poor effect on severe *C. psittaci* and should be combined with other drugs.

The intracellular activity of quinolones against *C. psittaci* was lower than that of tetracycline and macrolides, but they showed activity against *C. psittaci in vitro* (25, 26). In previous studies, 41 cases (54 cases in total) of severe patients had been treated with quinolones, among which 29 cases had failed to be treated with quinolones initially, and 6 cases were improved by quinolones alone. These results indicate that quinolones are effective in the treatment of *C. psittaci* and can be used for severe *C. psittaci* pneumonia, but there may be drug resistance in some patients and treatment failure. Currently, the use of antibiotics in the poultry and pet bird industries is increasing, which may have contributed to the drug resistance of *C. psittaci* (27). Our patient had been treated with cephalosporin and moxifloxacin intravenously for 7 days in the local hospital but did not improve. After being transferred to our department, the patient was given meropenem combined with levofloxacin intravenously but still had intermittent fever. *C. psittaci* in this patient was resistant to quinolones. After the diagnosis of *C. psittaci*, the patient was treated with tigecycline and improved. Tigecycline is a new tetracycline antibiotic that is mainly used for the treatment of severe abdominal infection, lung infection and bloodstream infection. There have been few reports on the treatment of Chlamydia psittacosis pneumonia with tigecycline. Wen et al. (28) reported a case of severe *C. psittaci* pneumonia in which tigecycline was used for treatment, but the treatment effect was not described in detail. Wu et al. (29) reported that three patients received carbapenems, linezolid, or tigecycline in addition to doxycycline treatment after diagnosis, but the outcomes were not stated. Kong et al. (30) reported that two patients with severe *C. psittaci* pneumonia were not effective in initial treatment with moxifloxacin and were improved after using tigecycline. This demonstrates that tigecycline can be used as an alternative treatment for severe *C. psittaci* pneumonia, especially in patients with coexisting secondary infections, because of its broad antibacterial spectrum. Whether tigecycline is better than other tetracycline drugs in severe patients needs further large-sample clinical observation.

In conclusion, the manifestations of *C. psittaci* pneumonia are diverse and lack specificity. The overall prognosis is good, but severe patients may die if they are not treated in time. The mNGS is a promising detection method. For patients with severe infections, patients who were not efficacious with empirical anti-infection treatment, or patients who were possibly infected with special pathogens, mNGS testing should be performed as soon as possible.

## Author contributions

YG contributed in diagnosing the disease, data collection, and data analysis. JL contributed to literature search and figures preparation. YG and JL were the main contributors to drafting the manuscript and performed the final manuscript review. Both authors read and approved the final manuscript.

## Abbreviations

mNGS: metagenomic next-generation sequencing
CT: computed tomography
BALF: bronchoalveolar lavage fluid
IL-6: interleukin-6
PCT: procalcitonin
CRP: C-reactive protein
FiO_2_: Fraction of inspired oxygen
pH: Pondus Hydrogenii
PaO_2_: partial arterial oxygen pressure
PaCO_2_: arterial partial pressure of carbon dioxide
PT: prothrombin time
APTT: activated partial thromboplastin time
ALT: alanine aminotransferase
CK-MB: creatine kinase isoenzyme
BNP: B-type natriuretic peptide
cTnI: cardiac troponin I
CEA: carcinoembryonic antigen
CYFRA: cytokeratin 19 fragment
NSE: neuron-specific enolase.
